# Knock Down of *Chlamydomonas reinhardtii* Phytyl Ester Synthase α Triggers DGAT3 Overexpression and Triacylglycerol Accumulation Under Low-Light Conditions

**DOI:** 10.3390/plants14193044

**Published:** 2025-10-01

**Authors:** Félix Eduardo Zegarra Borlando, Gerardo Martín Oresti, Natalia Pavia, María Verónica Beligni, Gabriela Gonorazky

**Affiliations:** 1 Instituto de Investigaciones Biológicas (IIB), Consejo Nacional de Investigaciones Científicas y Técnicas (CONICET), Universidad Nacional de Mar del Plata (UNMdP), Mar del Plata B7608FBY, Argentina; felixzegarra@mdp.edu.ar (F.E.Z.B.); nataliapavia@mdp.edu.ar (N.P.); 2 Instituto de Investigaciones Bioquímicas de Bahía Blanca (INIBIBB), Consejo Nacional de Investigaciones Científicas y Técnicas (CONICET), Universidad Nacional del Sur (UNS), Bahía Blanca B8000FWB, Argentina; gmoresti@criba.edu.ar; 3 Departamento de Biología, Bioquímica y Farmacia, Universidad Nacional del Sur (UNS), Bahía Blanca B8000FWB, Argentina

**Keywords:** *Chlamydomonas*, diacylglycerol acyltransferase, light response, phytol, phytyl ester synthase, triacylglycerols, fatty acid phytyl esters

## Abstract

Evidence indicates that light can trigger an increase in triacylglycerol (TAG) accumulation in eukaryotic microalgae without reducing cell division. In connection with this, we have recently reported that the expression of the chloroplast enzyme diacylglycerol acyltransferase 3 (DGAT3) is induced by light in concert with TAG accumulation in *Chlamydomonas reinhardtii*. In this work, we report the identification of two phytyl ester synthases (PES) in *C. reinhardtii,* named PESα and PESβ. These are homologous to chloroplast PES1 and PES2 of *Arabidopsis thaliana*, which play a role in the synthesis of fatty acid phytyl esters (FAPEs) and TAGs. We demonstrate that *PESα* and *PESβ* transcript levels are transiently induced upon transferring cell cultures from a growth condition of low light to high light, and this occurs in parallel to an increase in TAG levels. In a *pesα* knockdown mutant, *DGAT3* transcripts and TAG levels are significantly higher than in the parental strain at the end of the low-light period, and remain elevated after shifting *pesα* cells to the high-light condition. On the contrary, in a *pesβ* knockdown mutant, TAG levels, as well as DGAT3 expression, are similar to those of the control strain. These results suggest that PESα and PESβ are non-redundant in TAG metabolism and that PESα is functionally related to DGAT3.

## 1. Introduction

Eukaryotic microalgae are considered important oil producers [1]. One of the major lipid classes in these organisms are storage lipids in the form of triacylglycerols (TAGs) [1]. Nowadays, there is great interest in using native or engineered eukaryotic microalgae as a source of TAGs for biotechnological applications, such as biofuel and biopolymer manufacture [2]. In many species of microalgae, several stress conditions such as nutrient limitations induce TAG accumulation [1,3]. Nitrogen (N) starvation is the best characterized culture condition that stimulates TAG synthesis [1,3]. The enzymes triggered by this stress are mainly part of the conventional cytosolic/microsomal lipid synthesis pathway [4]. For industrial applications, the key challenge of such a scheme is that N depletion limits amino acid and protein synthesis, thereby reducing the rate of cell division and diminishing total lipid productivity [5]. For this reason, it is crucial to explore growth conditions that maximize TAG production without affecting cell growth.

*Chlamydomonas reinhardtii* is a unicellular green alga employed as an experimental model organism [6]. Goold et al. reported that high light induces TAG production in *C. reinhardtii* without affecting the rate of cell division [7]. Accordingly, it was observed that increasing light intensity across different spectral qualities boosts biomass concentration and neutral lipid accumulation in *C. reinhardtii* [8]. It was proposed that TAGs serve as key reservoirs for excess reducing power generated by the photosynthetic electron transport chain when energy input surpasses cellular utilization capacity, thereby mitigating photodamage [9]. The fact that high light can increase neutral lipid productivity without compromising cell growth, makes it a promising culture condition for the microalgal oil industry. However, the knowledge of non-conventional (i.e., non-microsomal) enzymatic pathways and their potential contribution to light-induced TAG synthesis remains limited.

Diacylglycerol acyltransferases (DGATs) are enzymes that catalyze the formation of TAGs by performing an acylation of an activated fatty acid onto the sn-3 position of a molecule of diacylglycerol [10]. The best characterized DGAT families are DGAT1 and DGAT2, which are membrane-bound enzymes of the cytosolic/microsomal pathway [10]. Interestingly, DGAT activity was also detected in chloroplast envelopes, though the specific isoform responsible for this activity remained unidentified [11]. Consistent with this finding, electron microscopy analysis revealed close associations between lipid droplets and both the endoplasmic reticulum and the outer membrane of the chloroplast envelope in *C. reinhardtii* and *Dunaliella bardawil* [12,13]. Given that DGAT1 harbors a predicted chloroplast transit peptide and is upregulated during senescence in *Arabidopsis thaliana* leaves—paralleling chloroplast TAG accumulation—this isoform is hypothesized to mediate lipid droplet formation at the chloroplast envelope [14,15].

In recent years, our group reported a DGAT exclusive to green algae, named DGAT3, which is moderately related to its plant homolog and structurally different from the canonical DGAT1 and DGAT2 families [15]. In silico analyses suggest that DGAT3 is a soluble protein that is targeted to the chloroplast [15,16]. Previous work showed that heterologous expression of *C. reinhardtii* DGAT3 in *Escherichia coli* cells increased the levels of TAGs in the presence of oleate, which indicates that DGAT3 has indeed DGAT activity [15]. We later reported that *DGAT3* transcripts were augmented in a *C. reinhardtii* wild-type strain upon transferring cell cultures from low light to high light [16]. Similar results were obtained in starch-deficient *C. reinhardtii* cells after shifting them from darkness to moderate light [16]. In both strains, the increase in *DGAT3* transcripts occurred in concert with a transient accumulation of TAGs [16]. In addition, we reported that DGAT1 expression was induced in *C. reinhardtii* cells, together with DGAT3, upon shifting cell cultures from low light to high light [16]. These results suggest that DGAT3 and DGAT1 play a role in chloroplast TAG production, and support the hypothesis about the existence of a non-conventional TAG synthesis pathway.

Phospholipid diacylglycerol acyltransferase (PDAT) transfers the acyl group from the sn-2 position of a phospholipid to the sn-3 position of DAG [10]. In *C. reinhardtii*, PDAT localizes to chloroplasts, with compelling evidence supporting its significant contribution to TAG biosynthesis under nitrogen-deprived conditions [15,17,18]. Furthermore, Chouhan et al. demonstrated that high light increases PDAT protein levels in *C. reinhardtii* cells in agreement with TAG accumulation [19]. Altogether, these results suggest that changes in light conditions might trigger the involvement of distinct enzymes in TAG production and point to the importance of further exploring these pathways.

Phytyl ester synthases (PESs) have been proposed as an alternative chloroplast TAG synthesis pathway in plants. Leaf senescence and stress conditions trigger the disintegration of the chloroplast membrane. This produces, among other metabolites, free phytol and free fatty acids (FFAs), which are derived from chlorophyll and lipid hydrolysis, respectively [20,21]. Both phytol and FFAs are cytotoxic metabolites and, consequently, must be rapidly recycled in order to avoid cell damage [22]. Thus, PESs provide a protective mechanism by catalyzing the acylation of a FFA onto a phytol molecule, resulting in the formation of fatty acid phytyl esters (FAPEs) [22]. Two PES isoforms were characterized in *A. thaliana*: PES1 (At1g54570) and PES2 (At3g26840), which belong to the estearase/lipase/thioestearase family [23]. Both proteins localize to the chloroplast and have an acyltransferase/DGAT domain [23,24,25]. In vivo and in vitro assays using heterologously expressed PES1 and PES2 demonstrated that both proteins generate FAPEs and TAGs [23]. Interestingly, the contents of both FAPEs and TAGs are strongly reduced in *pes1pes2* double mutants grown under N starvation [23]. Accordingly, heterologous expression of AtPES2 together with a Fatty Acid Reductase, which synthesizes fatty acid alcohols, and the transcription factor WRINKLED1, known to be involved in the regulation of fatty acid biosynthesis, enhanced wax esters and TAG accumulation in *Nicotiana benthamiana* [26]. From these results, it could be concluded that PES enzymes play a role in both FAPE and TAG synthesis in higher plants.

Thus far, FAPEs were detected in seven species of Chlorophyta and PES proteins were identified by proteomic analyses in the halotolerant alga *Dunaliella bardawil* [13,27,28]. This suggests that homologs of plant PES might exist in algae, which opens the possibility of their participation in FAPE and TAG synthesis.

In this work, we describe the identification of two PES isoforms in *C. reinhardtii*, named PESα and PESβ, homologous to PES1 and PES2 of *A. thaliana*. Our data reveal light-dependent upregulation of *PESα* and *PESβ* transcripts together with increased TAG levels in *C. reinhardtii* following transition from low-light to high-light conditions. Comparative analysis of *DGAT3*, *DGAT1*, and *PDAT* expression patterns, along with TAG accumulation profiles, showed significant differences between the *pesα* knockdown mutant and the parental strain. The putative role of PESα in non-conventional TAG synthesis is discussed.

## 2. Results

### 2.1. PESα and PESβ In Silico Analyses

**Figure 1:**
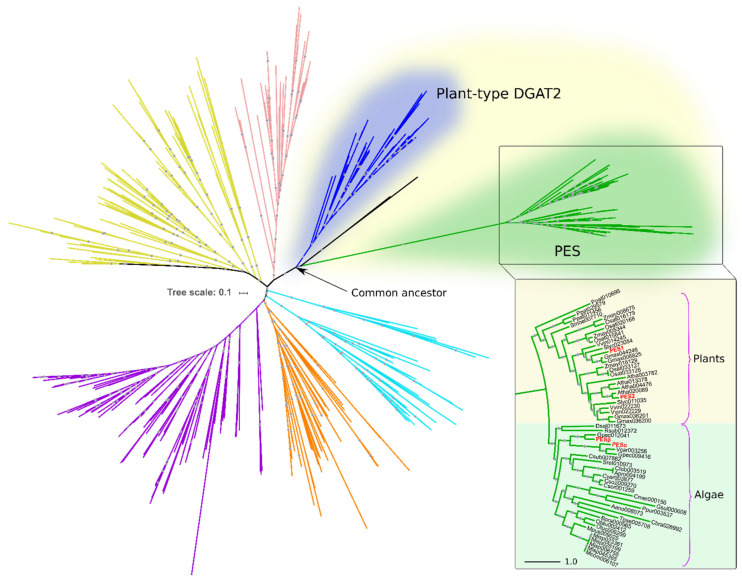
Algal and plant PES belong to the DAGAT superfamily. Rooted circular phylogram representation of the tree generated by the maximum likelihood (ML) method (500 bootstraps) on the proteins with DAGAT domain of the species detailed in Table S1. Gray circles represent ML bootstrap values > 50. Different colors represent distinct DAGAT protein clades previously reported by our group [15]. The inset shows the plant and algal PES clade. Numbers after the abbreviated species names are internal IDs. The names in red correspond to PES1 (At1g54570) and PES2 (At3g26840) from *A. thaliana*, and PESα (Cre08.g365950) and PESβ (Cre12.g521650) from *C. reinhardtii*. The scale bars represent the number of amino acid substitutions per site.

To identify PES homologs in algae, we conducted a HMMER iterative search using *A. thaliana* PES1 and PES2 as queries against predicted proteomes from algal species and from a set of representative eukaryotes (Table S1). The resulting candidates were then subjected to clustering and phylogenetic analyses, in order to establish their positioning within the superfamily of DAGAT domain-containing proteins (Pfam ID PF03982). Figure 1 shows the phylogenetic relationships of the DAGAT domain, present in DGAT2 and several related proteins, including PES.

The PES clade evolved from a most recent ancestor in common with plant DGAT2 (Figure 1). The sequences within this clade are restricted to photosynthetic, chlorophyll-containing, organisms. Among the species within our analysis, it includes ten plant species and nineteen species of green and red algae, showing a clear taxonomic division between them (Figure 1). PES1 and PES2 of *A. thaliana* reported by Lippold et al. are included within the plant PES group (Figure 1) [23]. In the algae group, we detected two PES sequences from *C. reinhardtii*, which we named PESα and PESβ (Figure 1). These are identified in Phytozome as genes Cre08.g365950 (https://phytozome-next.jgi.doe.gov/report/transcript/Creinhardtii_v5_6/Cre08.g365950.t1.2 (accessed on 9 September 2019)) and Cre12.g521650 (https://phytozome-next.jgi.doe.gov/report/gene/Creinhardtii_v5_6/Cre12.g521650 (accessed on 9 September 2019)), respectively.

PES1 and PES2 have very similar sequences, mainly differing in the 100 amino-terminal amino acids [23]. PESα and PESβ share 50% amino acid identity and 66% similarity with each other. Their most significant differences occur in the N-terminal regions, along with several distinct internal extensions present in both proteins. When compared to Arabidopsis PES proteins, PESα shows 33–34% identity and 48–49% similarity to PES1 and PES2 (Figure S1). PESβ exhibits slightly higher conservation, with 37% identity and 49–53% similarity to PES1 and PES2 (Figure S1).

**Figure 2:**
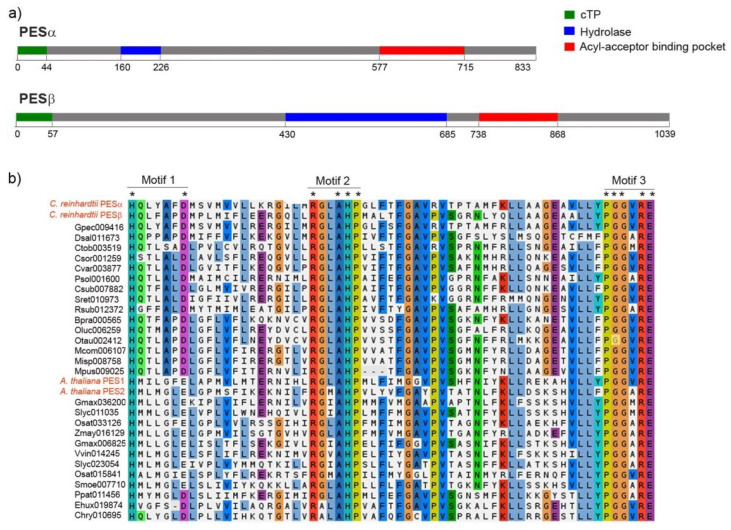
Protein sequence features of PESα and PESβ. (**a**) Sequence scanning with PredAlgo predicted that both, PESα and PESβ, have a chloroplast transit peptide (cTP, in green) of 44 and 57 amino acids, respectively. Conserved Domain Database analysis of PESα and PESβ revealed the presence of a hydrolase-like sequence (in blue: PESα: amino acids 160–226, PESβ: amino acids 430–685) and an acyl-acceptor binding pocket (in red: PESα: amino acids 577–715, PESβ: amino acids 738–868). (**b**) Multiple sequence alignment of the acyl-acceptor binding pocket motifs from PESα and PESβ, fifteen PES sequences from algae and fourteen PES sequences from plants, including PES1 and PES2 from *A. thaliana*. The alignment was performed with MAFFT using BLOSUM62 and default settings. The coloring reflects charge and polarity properties of the majority-rule consensus residues. The three characteristic motifs are shown. Asterisks indicate conserved residues. The *C. reinhardtii* and *A. thaliana* name tags are written in red.

The estimated molecular weight of PESα and PESβ is 94 kDa and 107 kDa, respectively, and this difference is mainly due to the presence of a longer amino-terminal extension in PESβ. Both proteins have predicted chloroplast localization, and the start of the mature proteins is presumed to be at positions Asp-45 and Gly-58 for PESα and PESβ, respectively (Figure 2a, green segments).

The in silico analysis of conserved domains revealed the presence of a predicted region of α/β hydrolase fold and a putative acyl acceptor-binding pocket (Figure 2a). In PESα, the α/β hydrolyase fold lies in the amino-terminal portion, while in PESβ it is situated centrally and is larger (Figure 2a, blue segments). In both sequences, the putative acyl acceptor-binding pocket is localized at the carboxy terminus (Figure 2a, red segments). According to this analysis, there are three motifs in the predicted acyl-binding pocket of *C. reinhardtii* PESs that are similar to acyltransferase motifs. These were detected by multiple sequence alignment of putative PES sequences from algal and plant species (Figure 2b). The residues histidine (H) and aspartic acid (D)/glutamic acid (E) of motif 1 are conserved in all the analyzed PES sequences, including PESα and PESβ (Figure 2b). The second motif is composed of strictly conserved arginine (R), alanine (A), H and proline (P) and two extra positions that correspond to conserved non-polar hydrophobic glycine (G)/A and leucine (L)/isoleucine (I)/valine (V)/methionine (M) residues (Figure 2b). The third motif is formed by strictly conserved P, two G, R and E and a conserved position with non-polar hydrophobic V/A/M residues (Figure 2b).

According to in silico predictions, PESα and PESβ do not have transmembrane regions, which suggests that both are soluble proteins. Hydrophobicity analysis indicated that PESα and PESβ have 46.6% and 54% hydrophobic amino acid residues, respectively, which is consistent with their likely association to membranes and interaction with lipid substrates.

### 2.2. Analysis of PESα and PESβ Transcripts in the Parental Strain, cc-5325, After Shifting Cells from Low Light to High Light

RNA-seq results reported by Wittkopp et al. showed that the expression of *PESα* and *PESβ* is rapidly and transiently induced in *C. reinhardtii* cells by changes in the light conditions [29]. Based on this evidence, we hypothesized that PESα and PESβ could play a role in light-induced lipid synthesis. With the purpose of evaluating this assumption, we first examined the response of *PESα* and *PESβ* to light. We cultured the cc-5325 strain of *C. reinhardtii* on Tris-acetate-phosphate (TAP) medium under continuous illumination (50 µmol photon m^−2^ s^−1^) until reaching exponential phase (2 × 10^6^ cells mL^−1^). Then, the cells were transferred to low light (14 µmol photon m^−2^ s^−1^) for 16 h and subsequently shifted to high light (140 µmol photon m^−2^ s^−1^) for 1, 4 and 7 h. Expression was determined by reverse transcription quantitative polymerase chain reaction (RT-qPCR). The *Chlamydomonas Guanine nucleotide-binding protein subunit β-Like Polypeptide* (*GBLP*) gene was used as an endogenous control, since its transcript levels were previously demonstrated to remain stable during dark/low-light to high-light transitions [16]. Transcript levels were analyzed in relation to the End of the Low Light (ELL) period.

**Figure 3:**
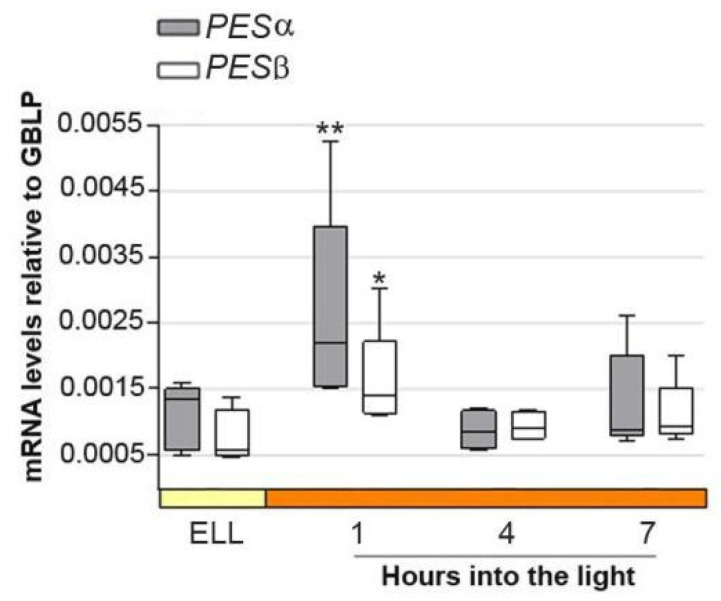
*PESα* and *PESβ* mRNAs increase in cc-5325 after shifting cells from low light to high light. Cells from the parental cc-5325 strain were grown in TAP medium under continuous light (50 µmol photon m^−2^ s^−1^) to approximately 2 × 10^6^ cells mL^−1^, then transferred to 14 µmol photon m^−2^ s^−1^ (low light period) for 16 h and subsequently switched to 140 µmol photon m^−2^ s^−1^ (high light). Samples were harvested at the end of the low-light period (ELL) and at 1, 4 and 7 h after transferring the cells to high light. Total RNAs were extracted from each sample and mRNAs were analyzed by RT-qPCR. *GBLP* was used as endogenous control. Box plots show the expression of *PESα* and *PESβ*. The pale yellow box and the orange box indicate the ELL and high-light periods, respectively. Results are expressed as mRNA levels relative to *GBLP*. Vertical bars indicate minimum and maximum values and horizontal black strips indicate median values (n = 5). Asterisks indicate significant difference from the corresponding ELL sample according to one-way ANOVA, post hoc Dunnett’s: ** *p* < 0.05. * *p* < 0.1.

Figure 3 shows that *PESα* and *PESβ* transcript levels peaked 2.4 and 2.6-fold, respectively, as early as 1 h into the high-light period, and then decreased at 4 h and 7 h. This result suggests that *PESα* and *PESβ* are light-responsive genes.

### 2.3. Molecular Characterization of Pesα and Pesβ Mutants

**Figure 4:**
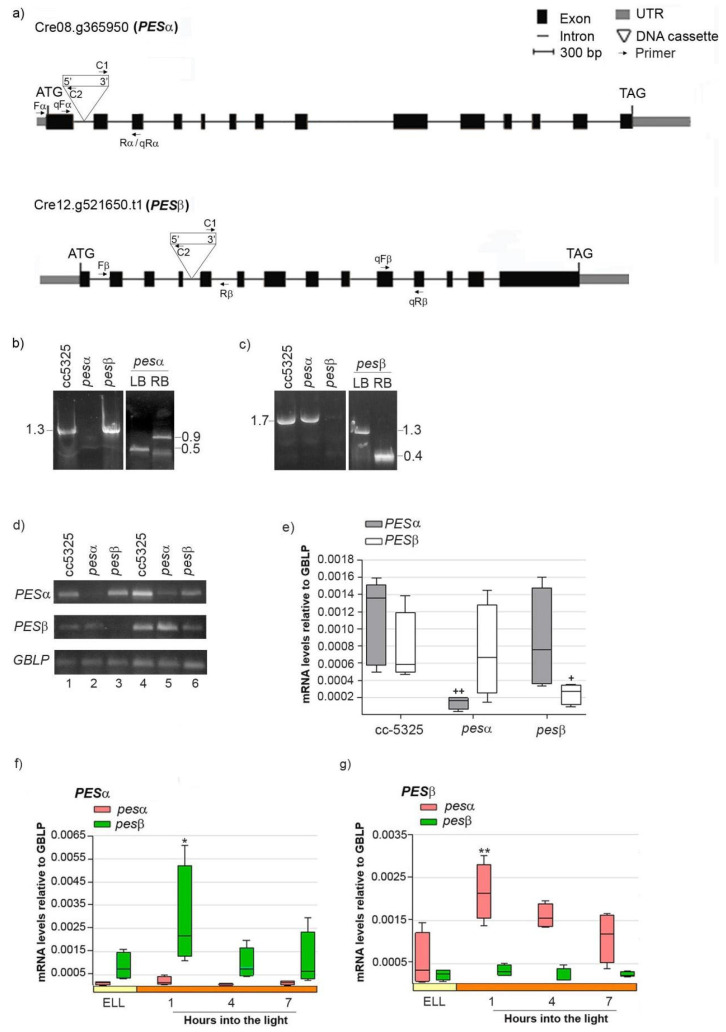
Molecular characterization of *C. reinhardtii pesα* and *pesβ* mutants. (**a**) Exon/intron structure and location of the DNA cassette insertion in genes Cre08.g365950 (*PESα*) and Cre12.g521650 (*PESβ*). UTRs, exons and introns are designated by gray boxes, black boxes and lines, respectively. Binding sites for primers employed in the PCR with genomic DNA analysis Fα (Forward PESα), Rα (reverse PESα), Fβ (Forward PES β), Rβ (Reverse PESβ), C1 (Forward DNA cassette) and C2 (Reverse DNA cassette), are indicated, as well as the binding sites for primers employed in the semi-quantitative and quantitative RT-PCR analysis qFα (qPCR Forward PESα), qRα (qPCR reverse PESα), qFβ (qPCR Forward PES β), qRβ (qPCR Reverse PESβ). (**b**) PCR using genomic DNA of cc-5325, *pesα* and *pesβ* and the primer pair “Fα + Rα” to amplify *PESα* (1.3 kb, left panel). Confirmation of *PESα* disruption by PCR with genomic DNA of *pesα* and the primer pairs “Fα + C2” and “C1 + Rα”, to amplify the left border (LB, 0.5 kb) and right border (RB, 0.9 kb) of the DNA cassette insertion, respectively (right panel). (**c**) PCR with genomic DNA of cc-5325, *pesα* and *pesβ* and the primer pairs “Fβ + Rβ” to amplify *PESβ* (1.7 kb, left panel). Confirmation of *PESβ* disruption by PCR with genomic DNA of *pesβ* and the primer pairs “Fβ + C2” and “C1 + Rβ”, to amplify the left border (LB, 1.3 kb) and right border (RB, 0.4 kb) of the DNA cassette insertion, respectively (right panel). (**d**–**g**) Expression of PESα and *PESβ* in the parental and mutant backgrounds: Cells from cc-5325, *pesα* and *pesβ* strains were grown in TAP medium under continuous light (50 µmol photon m^−2^ s^−1^) to approximately 2 × 10^6^ cells mL^−1^, then transferred to 14 µmol photon m^−2^ s^−1^ (low-light period) for 16 h and subsequently switched to 140 µmol photon m^−2^ s^−1^ (high light). Samples were harvested at the end of the low light period (ELL) and at 1, 4 and 7 h after transferring the cells to high light. (**d**) Semi-quantitative RT-PCR of *PESα* and *PESβ* using RNAs from the ELL samples of cc-5325, *pesα* and *pesβ* mutants. *GBLP* was used as endogenous control. The PCR conditions were: 35 or 40 cycles for *PESα* (upper panel, lanes1–3 and 4–6, respectively); 30 or 33 cycles for *PESβ* (middle panel, lanes 1–3 and 4–6, respectively) and 20 or 23 cycles for the endogenous control, *GBLP* (lower panel, lanes 1–3 and 4–6, respectively). (**e**) RT-qPCR using ELL samples of cc-5325, *pesα* and *pesβ*. Box plots show mRNA levels of *PESα* and *PESβ* normalized to *GBLP*. (**f**,**g**) Expression of *PESα* and *PESβ* in *pesα* and *pesβ* cells following a transition from low light to high light analyzed by RT-qPCR. Results are expressed as mRNA levels relative to *GBLP*. The pale yellow box and the orange box indicate the ELL and high-light periods, respectively. Semi-quantitative RT-PCR and RT-qPCR were performed with the primer pairs “qFα + qRα” and “qFβ + qRβ” to amplify *PESα* and *PESβ*, respectively. Vertical bars indicate minimum and maximum values and horizontal black strips indicate median values (n = 5). Significant differences between cc-5325 and *pesα*/*pesβ* mutants are indicated by plus symbols (+), while significant differences between ELL and high-light conditions within each strain are indicated by asterisks (*) according to one-way ANOVA, post hoc Dunnett’s: ^++^ ** *p* < 0.05, ^+^ * *p* < 0.1.

In order to study if PESα and PESβ participate in a light-induced lipid synthesis pathway, we acquired mutants from the Chlamydomonas Library Project (CLiP, https://www.chlamylibrary.org/ (accessed on 4 April 2020)). This is a collection of *C. reinhardtii* strains generated by random insertion of a 2.2 kb paromomycin-resistance cassette into the cc-5325 strain [30]. We selected the lines LMJ.RY0402.052315 (*pesα*) and LMJ.RY0402.046050 (*pesβ*), which contain the DNA cassette inserted in intron 1 and intron 4 of the *PESα* and *PESβ* genes, respectively, based on a 95% mapping confidence (Figure 4a) [30].

To confirm that the insertions were indeed present in the *PESα* and *PESβ* genes, we mapped the location of each insertion by PCR using oligonucleotides specific to both the genes and the insertion sites. Figure 4b (left panel) shows that, in the cc-5325 and *pesβ* strains, we obtained a PCR product of 1.3 kb that spans the *PESα* insertion site, whereas this product was not observed when using the genomic DNA of *pesα* as template. The insertion site within the first intron of *PESα* was verified by amplifying the left border (LB, 0.5 kb) and right border (RB, 0.9 kb) junctions of the DNA cassette (Figure 4b, right panel).

A 1.7 kb amplicon spanning the *PESβ* insertion site was obtained from the cc-5325 and *pesα* strains, but not from *pesβ* (Figure 4c, left panel). We confirmed the disruption of the fourth intron of *PESβ* by amplifying the LB (1.3 kb) and RB (0.4 kb) junctions of the DNA cassette (Figure 4c, right panel). Semi-quantitative RT-PCR detected PES*α* transcript expression in cc-5325 and *pesβ* at 35 PCR cycles, while they were only detectable at 40 PCR cycles in *pesα* (Figure 4d, upper panel). Similarly, *PESβ* mRNAs were detected in cc-5325 and *pesα* at 30 PCR cycles, while these were only detectable at 33 PCR cycles in *pesβ* (Figure 4d, middle panel). *GBLP* was equally amplified in the three strains at 20 and 23 PCR cycles, indicating that equivalent amounts of RNA were used in the semi-quantitative RT-PCR analysis. Therefore, these results suggest that *pesα* and *pesβ* are knockdown mutants rather than complete knockouts. Quantification of *PESα* and *PESβ* transcripts by RT-qPCR revealed detectable mRNA levels in the respective *pesα* and *pesβ* mutants, though significantly reduced compared to the parental strain (Figure 4e). In contrast, *PESα* mRNAs in *pesβ* and *PESβ* mRNAs in *pesα* were similar to those in cc-5325 (Figure 4e). This confirms the results obtained by semi-quantitative RT-PCR.

Next, we evaluated the light-dependent expression of *PESα* and *PESβ* in the *pesα* and *pesβ* mutant backgrounds, as previously performed with the parental strain. Analysis by RT-qPCR revealed that *PESα* was not induced in *pesα*, nor was *PESβ* in *pesβ* cells (Figure 4f,g). Conversely, *PESα* and *PESβ* transcripts were transiently increased in the *pesβ* and *pesα* strains, respectively, at 1 h into the high-light period (Figure 4f,g). The induction levels were similar to those observed in cc-5325 (Figure 3). These results suggest that the high-light induction of *PESα* and *PESβ* mRNAs is not affected by the mutation of the alternative isoform.

To further investigate the phenotype of *pesα* and *pesβ* mutants, we analyzed seven-day growth curves and the cell areas of cc-5325, *pesα* and *pesβ* cells cultured under standard conditions. Figure S2a shows that the growth curves of cc-5325, *pesα* and *pesβ* were similar. A transient increase in cell density was detected in the *pesβ* mutant at day five, but comparable cell densities were reached at the end of the exponential phase. Consistent with this result, analysis of growth rates revealed no significant differences between any of the strains (Figure S2b). This suggests that knockdown of *PESα* and *PESβ* does not impair normal growth.

In contrast, analysis of cell morphology revealed an altered phenotype for the *pesα* mutant. These cells exhibited a significantly larger cell area during the exponential growth phase and a strong tendency to form palmelloids (i.e., non-dividing cell aggregates), a typical algal stress response (Figure S2c,d) [31]. Conversely, morphological analysis of the *pesβ* mutant revealed only a significantly smaller cell area on the fourth day of growth, a phenotype that was not sustained by the fifth day.

### 2.4. Analysis of DGAT3, DGAT1, PDAT Expression and TAG Levels in cc-5325, Pesα and Pesβ Strains After Shifting Cells from Low Light to High Light

Substantial evidence suggests that DGAT3, DGAT1 and PDAT are chloroplast enzymes involved in TAG synthesis [11,14,15,16,18]. In contrast, DGAT2, which is encoded by a family of six isoforms, functions exclusively in microsomal TAG synthesis [15]. Because *PESα* and *PESβ* are predicted to localize to the chloroplast, we investigated whether they are related to DGAT3, DGAT1 and PDAT.

**Figure 5:**
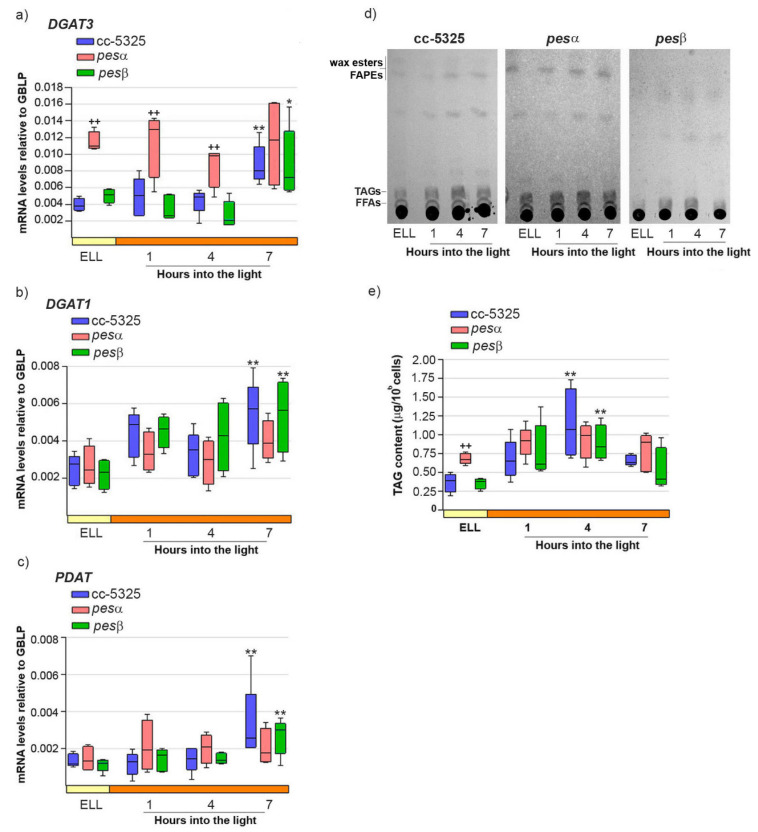
Response of *DGAT3*, *DGAT1*, *PDAT* mRNAs and TAGs upon shifting cc-5325, *pesα* and *pesβ* cells from low light to high light. Cells from the parental cc-5325 (control), *pesα* and *pesβ* strains were grown in TAP medium under continuous light (50 µmol photon m^−2^ s^−1^) to approximately 2 × 10^6^ cells mL^−1^, then transferred to 14 µmol photon m^−2^ s^−1^ (low-light period) for 16 h and subsequently switched to 140 µmol photon m^−2^ s^−1^ (high light). Samples were harvested at the end of the low light period (ELL) and at 1, 4 and 7 h after transferring the cells to high light. (**a**–**c**) Total RNAs were extracted from each sample and specific mRNAs were analyzed by RT-qPCR. *GBLP* was used as endogenous control. Box plots show the expression of *DGAT3* (**a**), *DGAT1* (**b**) and *PDAT* (**c**) in cc-5325, *pesα* and *pesβ* strains. Results are expressed as mRNA levels relative to *GBLP*. (**d**) Total lipids were extracted from each sample and analyzed by thin-layer chromatography (TLC). Sample volumes equivalent to 35 million cells were loaded on each lane. Dashes show the spots of free fatty acid (FFA), triacylglycerol (TAG) and wax ester/FAPE standards. A representative image of five independent biological replicates is shown. (**e**) TAGs eluted from the spots on (**d**) were quantified using a colorimetric assay. Box plots show the content of TAGs in cc-5325, *pesα* and *pesβ* strains. The pale yellow box and the orange box indicate the ELL and high-light periods, respectively. Vertical bars indicate minimum and maximum values and horizontal black strips indicate median values (n = 5). Significant differences between cc-5325 and *pesα*/*pesβ* mutants are indicated by plus symbols (+), while significant differences between ELL and high-light conditions within each strain are indicated by asterisks (*) according to one-way ANOVA, post hoc Dunnett’s: ^++^ ** *p* < 0.05, * *p* < 0.1.

Our previous work demonstrated that high-light conditions upregulate both *DGAT3* and *DGAT1* mRNA levels while concurrently increasing TAG accumulation in *C. reinhardtii* [16]. We also proposed a functional role for PDAT in this stress response [16]. Therefore, we analyzed whether the expression pattern of DGAT3, DGAT1 and PDAT, as well as TAG content, were modified in *pesα* and *pesβ* strains. Figure 5a–c shows that *DGAT3*, *DGAT1* and *PDAT* mRNAs were significantly increased in cc-5325, 7 h after shifting cells from low light to high light.

In the same conditions, TAGs reached a 3.1-fold increase in cc-5325 cells at 4 h into the high-light period, and decreased at 7 h (Figure 5d,e).

In the *pesα* mutant, *DGAT3* transcript levels were 2.9-fold higher than in cc-5325 at the ELL condition, and remained elevated throughout the subsequent high-light period (Figure 5a). In line with *DGAT3* expression, the *pesα* strain had 1.9-fold more TAGs than cc-5325 at ELL (Figure 5d,e). The higher TAG content in *pesα* at ELL remained at similar levels until the end of the experiment, consistent with our observations of *DGAT3* transcripts. These results demonstrate that the *pesα* knockdown strain exhibits elevated *DGAT3* expression and increased TAG accumulation prior to high-light exposure compared to the parental strain. Consistent with this result, we observed that *pesα* cells had an enlarged cell morphology that was similar to the phenotype shown in Supplemental Figure S2c,d.

In contrast to *DGAT3*, both *DGAT1* and *PDAT* transcript levels were comparable in *pesα* and cc-5325 strains in the ELL condition. Notably, neither gene showed significant upregulation in *pesα* following the transition to high-light conditions (Figure 5b,c). Thus, it could be concluded that *PESα* knockdown affects the expression of *DGAT3*, *DGAT1*, and *PDAT* differently.

In the *pesβ* mutant, *DGAT3*, *DGAT1*, and *PDAT* exhibited expression patterns similar to cc-5325, showing 2–3 fold induction within 7 h following the transfer from low-light to high-light conditions (Figure 5a–c). TAG levels were transiently augmented at 4 h after switching *pesβ* cells from low light to high light, as observed in cc-5325 (Figure 5d,e). No morphological differences with the control were observed under high-light conditions, in concert with a lack of increased TAG accumulation. These results suggest that *PESβ* knockdown does not affect the high-light induction of *DGAT3*, *DGAT1*, and *PDAT* mRNAs, nor the content of TAGs.

### 2.5. Analysis of FAPEs, Phytol and Chlorophyll Levels in cc-5325, Pesα and Pesβ Strains upon Shifting Cells from Low Light to High Light

**Figure 6:**
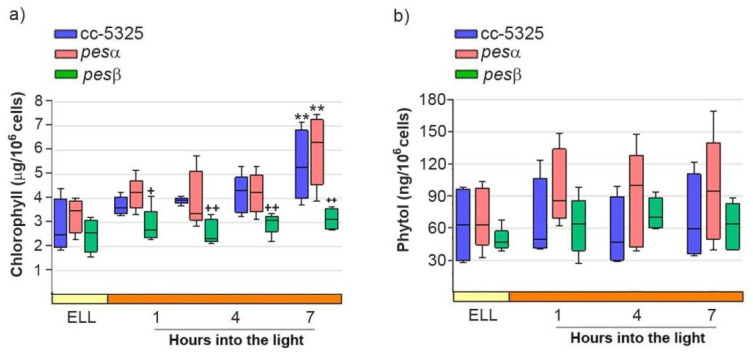
Analysis of chlorophyll and phytol content after shifting cc-5325, *pesα* and *pesβ* cells from low light to high light. Cells from the parental cc-5325 (control), *pesα* and *pesβ* strains were grown in TAP medium under continuous light (50 µmol photon m^−2^ s^−1^) to approximately 2 × 10^6^ cells mL^−1^, then transferred to 14 µmol photon m^−2^ s^−1^ (low-light period) for 16 h and subsequently switched to 140 µmol photon m^−2^ s^−1^ (high light). Samples were harvested at the end of the low-light period (ELL) and at 1, 4 and 7 h after transferring the cells to high light. (**a**) Chlorophyll was extracted from each sample and measured photometrically. Box plots show the content of chlorophyll in cc-5325, *pesα* and *pesβ* strains. (**b**) Total lipids were extracted from each sample and separated by TLC. Sample volumes equivalent to 35 million cells were loaded on each lane. Phytol spots were eluted from the silica of the TLC plates and quantified by HPLC. Box plots show the content of phytol in cc-5325, *pesα* and *pesβ* strains. The pale yellow box and the orange box indicate the ELL and high-light periods, respectively. Vertical bars indicate minimum and maximum values and horizontal black strips indicate median values (n = 5). Significant differences between cc-5325 and *pesα*/*pesβ* mutants are indicated by plus symbols (+), while significant differences between ELL and high-light conditions within each strain are indicated by asterisks (*) according to one-way ANOVA, post hoc Dunnett’s: ^++^ ** *p* < 0.05, ^+^ *p* < 0.1.

We further analyzed FAPE levels in the cc-5325, *pesα* and *pesβ* strains upon shifting the cells from low light to high light. Given that FAPEs and wax esters co-migrate in TLC, we isolated the wax ester/FAPE fractions from the TLCs shown in Figure 5d [32,33]. We then attempted to identify and quantify FAPEs in these fractions using gas chromatography-mass spectrometry (GC-MS). No detectable FAPEs were found in any of the strains grown under both low light and high light. Since FAPE synthesis depends on free phytol produced from chlorophyll degradation, we further analyzed chlorophyll and phytol content in cc-5325, *pesα* and *pesβ* cells grown under both low-light and high-light conditions. As shown in Figure 6a, chlorophyll content did not differ significantly between the parental cc-5325 strain and the *pesα* mutant throughout the experimental period. Following 7 h of exposure to high light, chlorophyll levels increased significantly in both cc-5325 and *pesα*. In contrast, *pesβ* showed significantly lower chlorophyll levels compared to cc-5325 and *pesα* during the high-light period.

Figure 6b shows that phytol levels remained stable in cc-5325 following the transition from low light to high-light conditions and throughout the whole experimental period. Neither *pesα* nor *pesβ* showed significant differences in phytol content compared to the parental strain under either light regime (Figure 6b). This indicates that, under the experimental conditions tested, there is no significant chlorophyll degradation and, hence, phytol does not accumulate following high-light exposure.

## 3. Discussion

In this report, we identify two PES isoforms in *C. reinhardtii*, named PESα and PESβ. We demonstrate that *PESα* and *PESβ* are transiently induced by high light, consistent with an increase in TAG levels. Interestingly, *pesα* cells accumulate TAGs and upregulate *DGAT3* transcripts—a gene proposed to be involved in light-dependent TAG synthesis—even under low-light conditions, prior to exposure to high light. Moreover, in *pesα* cells, *DGAT1* and *PDAT* are not induced by high light, unlike in parental cells. This phenotype is absent in *pesβ* cells, where TAG levels and *DGAT3*, *DGAT1* and *PDAT* transcript levels remain similar to those of control cells. These findings suggest that PESα and PESβ are functionally non-redundant in TAG metabolism and implicate PESα as a novel component of a non-conventional, light-induced, TAG synthesis pathway in *C. reinhardtii*, likely alongside DGAT3, DGAT1 and PDAT.

High light induces lipid accumulation in various algal species, directing excess energy toward chemical products and preventing photooxidative damage [34]. Under this growth condition, it was observed that TAGs are mainly of chloroplastic origin and accumulate in lipoproteic particles called lipid droplets, mostly associated with the chloroplast envelope [7]. Chloroplastic TAGs can also accumulate in plastoglobules, which are lipid droplets that are associated with thylakoids and proliferate in plants during senescence and oxidative stress conditions [35]. PES1 and PES2 have been described as proteins associated with plastoglobules that play a role in recycling chlorophyll and lipids derived from the chloroplastic membrane, by producing FAPEs and TAGs [23,24,25]. Our data mining analysis identified PESα and PESβ in *C. reinhardtii* as homologs of Arabidopsis PES1 and PES2, consistent with previous phylogenetic analyses [13,23,33,36].

Both, PESα and PESβ, have a putative hydrolase domain and an acyl-binding pocket, which are characteristic of acyltransferase proteins such as PES1 and PES2 [23]. The α/β hydrolase fold is common to several hydrolytic enzymes of widely differing phylogenetic origin and catalytic function [37]. The presence of this domain suggests that PESα and PESβ may have lipase activity, as previously postulated for PES1 and PES2 [23]. Regarding the putative acyl-binding pocket of PESα and PESβ, the first motif—containing the conserved H and D/E residues—represents a critical component of the acyltransferase catalytic domain and has been previously identified in DGAT3 [15,38]. PESα and PESβ are predicted to be hydrophobic proteins without transmembrane regions, as previously described for PES1 and PES2 in the proteomic analysis of Arabidopsis plastoglobules [25]. Their hydrophobicity suggests that PESα and PESβ could interact with membranes or in general with lipid compounds, as it has been proposed for DGAT3 [15].

The sequences of PESα and PESβ proteins differ in their N-terminal portions, in which both isoforms have specific extensions, being the one of PESβ longer than that of PESα. It has been reported that plant and algal proteins involved in light-dependent reactions are regulated by distinct post-translational modifications and by the binding of other proteins, which affect activity, stability, localization and other characteristics of the protein fate [39]. In this scenario, we could argue that the differences in the N-terminal portions of PESα and PESβ could provide different opportunities for post-translational regulation.

PESα and PESβ are predicted to have chloroplastic localization, as recently reported by Wang et al. [40]. Accordingly, Davidi et al. detected three PES proteins in plastoglobule fractions of *D. bardawil* that are homologs of PESα and PESβ and have similar predicted molecular weights [13].

We demonstrate that *PESα* and *PESβ* mRNAs are transiently increased shortly after shifting cc-5325 cells from low light to high light conditions. This pulse pattern is consistent with light activation, in which the transient response is thought to meet the immediate requirements of photosynthetic activity following such a shift. As the light period progresses, these requirements diminish as cells adapt through a light-dependent genetic program. In *C. reinhardtii*, this response is regulated mainly at the transcriptional and/or RNA stability level, particularly in chloroplast-targeted genes such as *DGAT3*, which are functionally linked to photosynthetic activity [16,41,42,43,44,45,46]. Additionally, this is consistent with RNA-seq results showing that *PESα* and *PESβ* expression is rapidly and transiently induced in *C. reinhardtii* within 0.5–1 h after transferring cells from dark to high light [29,42]. Similarly, it has been reported that Arabidopsis PES1 protein levels and tomato (*Solanum lycopersicum*) *PES2* mRNAs increase in plants grown under high light [25,47]. Despite this evidence, little is known about the mechanisms that regulate *PES* expression. RNA-seq analysis revealed that biliverdin—a product of the heme catabolism involved in plastid-to-nucleus retrograde signaling in plants—attenuates the light-induced expression of *PESα* and *PESβ* in *C. reinhardtii* [29]. Additionally, Kamranfar et al. reported that *PES1* is regulated by the transcription factor RD26, which is involved in metabolic reprogramming during senescence [48]. Future work will be focused on studying the regulatory mechanisms of *PESα* and *PESβ*.

We previously proposed that *DGAT3* and *DGAT1* are induced in cc-125 wild-type cells when light intensity is increased, which occurs in concert with an increase in TAG content [16]. We obtained similar results in cc-5325 cells, from which we conclude that these light-dependent responses are reproducible in both *C. reinhardtii* strains. In order to analyze the roles of PESα and PESβ in the light-dependent lipid synthesis pathway, we acquired two insertional mutants, *pesα* and *pesβ*, which we characterized as knockdown mutants. In this type of transformants, a knockdown mutation may result from a small population of mRNAs in which the entire insertion cassette is spliced out together with the flanking intron sequence, leading to considerably lower, but not null, mRNA expression, as previously reported [49,50]. Despite this, we observed that the mutated genes are not induced by high light, as are the wild-type genes, and that the mutation of one PES isoform does not affect the expression of the other. We show, as well, that *DGAT3* mRNAs were significantly higher in *pesα* cells than in the parental strain in the ELL sample, and this was maintained along the high-light period. Interestingly, we observed that *pesα* cells had higher TAG levels than the parental strain under the same growth condition. These results support our hypothesis regarding a role of DGAT3 in TAG synthesis in *C. reinhardtii*, as DGAT3 overexpression coincided with an increase in TAG content in *pesα* cells. The upregulation of DGAT3 in *pesα* cells may be attributed to genetic compensation, a mechanism observed in the cellular networks of various eukaryotic species. In this process, the disruption of a specific gene can lead to changes in the expression of other genes within the same network, in order to preserve cellular viability [51]. Specifically, Lee et al. reported that DGAT2-2 knockout triggered the overexpression of *DGAT2-1*, *DGAT2-3* and *PDAT* in P-deprived *C. reinhardtii* cultures [52]. In contrast, *DGAT2-1* knockout only induced *PDAT* overexpression [52]. In both cases, this response occurred in concert with the overaccumulation of TAGs [52]. The authors concluded that compensating for mutations in genes involved in key steps of lipid biosynthesis with related genes mitigates their impact, thereby enhancing the genetic robustness of microalgal TAG production [52]. We hypothesize that a similar mechanism may apply to PESα and DGAT3, where *DGAT3* overexpression compensates for *PESα* knockdown, resulting in TAG over-accumulation under low-light conditions. This suggests that PESα and DGAT3 are functionally related genes involved in non-conventional TAG synthesis. In contrast to the results observed in *pesα* cells, the *A. thaliana pes1pes2* double mutant, along with the PES-related mutants *slr2103* from *Synechocystis* sp. PCC 6803 and *A0918* from *Synechococcus* sp. PCC 7002, exhibited reduced TAG levels compared to their respective wild-type strains [23,33,36]. Since these were null mutants, it could be hypothesized that: (i) the combined mutation of both *PES1* and *PES2* creates a different and/or more severe phenotype or (ii) distinct phenotypes arise from knockout versus knockdown PES strains. Further studies will be performed in order to analyze TAG production in a *pesα* knockout background, as well as in *pesαpesβ* double mutants.

Unlike *DGAT3*, the levels of *DGAT1* and *PDAT* mRNAs in *pesα* cells under low-light conditions were comparable to those in the parental strain, but did not increase when exposed to high light. This suggests that PESα is necessary, either directly or indirectly, for the high-light dependent expression of *DGAT1* and *PDAT*. Alternatively, the elevated levels of TAGs in the *pesα* background might inhibit the expression of *DGAT1* and *PDAT* induced by light, as we previously proposed [16].

In contrast to the *pesα* phenotype, *pesβ* knockdown had no effect on the expression of *DGAT3*, *DGAT1*, or *PDAT*, nor on TAG content under both low-light and high-light conditions. Overall, these data suggest that PESα and PESβ are likely non-redundant isoforms in TAG metabolism, and that *PESβ* does not have a noticeable role in light-dependent TAG accumulation.

It has been postulated that FAPEs serve as a temporary storage for free phytol and free fatty acids, which are released from chlorophyll and galactolipids during chlorotic stress and later recycled when the stress subsides [22]. No FAPEs were detected in cc-5325 in our culture conditions, consistent with the lack of observable chlorophyll degradation or phytol accumulation in any of the strains across both light regimes. Plants grown under standard conditions contain very few FAPEs, but their levels significantly increase under chlorotic stresses, such as senescence and nitrogen deprivation, in concert with chlorophyll decrease and phytol increase [23,32,53,54]. Conversely, Spicher et al. showed that only two out of nine FAPE species, 14:0-phytol and 16:0-phytol, are increased by high light (850 µmol m^−2^ s^−1^) in tomato plants, while phytol levels remain constant [47]. These reports suggest that FAPEs are primarily produced under conditions that induce massive chlorophyll degradation and increased phytol levels. In addition, it has been demonstrated that chlorophyll levels do not change significantly in *C. reinhardtii* cells grown for up to 48 h at 500 µmol m^−2^ s^−1^ [55,56]. These findings support our results, which show that the light intensities used in our assays did not induce substantial chlorophyll degradation. This could explain the lack of phytol accumulation and, consequently, the absence of FAPEs in *C. reinhardtii* cells.

These results further suggest that, during low- to high-light transitions, *PESα* functions primarily in the TAG synthesis pathway, with no detectable role on FAPE accumulation.

## 4. Materials and Methods

### 4.1. Chlamydomonas reinhardtii Strains and Culture Conditions

The *C. reinhardtii* mutant strains LMJ.RY0402.052315 (*pesα*) and LMJ.RY0402.046050 (*pesβ*), as well as the parental strain cc-5325 (*cw15 mt-*), were obtained from the Chlamydomonas Resource Center (https://www.chlamycollection.org). The mutant strains were generated by the Chlamydomonas Library Project (CLiP, https://www.chlamylibrary.org/) by random insertion of a 2.2 kb paromomycin-resistance cassette named CIB1 into cc-5325 cells [30]. The strains were maintained on TAP agar plates [57]. For experiments, the cells were inoculated at 10^4^ cells mL^−1^ in 100 mL Erlenmeyer flasks containing liquid TAP. The cultures were grown on a rotary shaker at 21 °C under continuous white light (6500 K) at 50 µmol photon m^−2^ s^−1^ until they reached a cell density of 2 × 10^6^ cells mL^−1^. Then, they were transferred to low light (14 µmol photon m^−2^ s^−1^) for 16 h and subsequently shifted to high light (140 µmol photon m^−2^ s^−1^). Cell cultures were grown in batch mode until the end of the low light period and in semi-continuous mode after transferring them to the light [16]. Samples were taken at the ELL and then 1, 4 and 7 h after shifting the cultures to high light. Cell harvesting was performed as previously described [16]. Five independent experiments were conducted for each culture condition and strain.

### 4.2. Determination of Growth Curve, Growth Rate and Cell Area

Aliquots from cultures of cc-5325, *pesα*, and *pesβ* grown at 50 µmol photon m^−2^ s^−1^ were harvested at 3, 4, 5, 6, and 7 days of growth, thereby covering the lag, exponential, and stationary phases. Cell density was determined by cell counting using an Olympus BH2 microscope (Olympus Corporation of the Americas, Breinigsville, PA, USA) and a hemocytometer. Cell density values were plotted against time, and the growth rate (µ) was calculated according to the following formula [58]:

µ = (ln(x_2_) − ln(x_1_))/(t_2_ − t_1_),

where “x_2_” and “x_1_” correspond to the cell densities recorded on day 6 (t_2_, end of the exponential phase) and day 3 (t_1_, beginning of the exponential phase), respectively.

To determine cell area, cells from the three strains harvested at 4 and 5 days of growth were visualized using an Olympus CKX53 microscope (Olympus Corporation of the Americas, Breinigsville, PA, USA) and digitized with an AmScope MU1000 camera (United Scope LLC, Irvine, CA, USA) attached to the microscope. Cell area was measured using the ImageJ software (version 1.54g, Wayne Rasband, NIH, Bethesda, MD, USA).

### 4.3. In Silico Analyses

PES homologs were identified with the phmmer tool within HMMER version 3 using *A. thaliana* PES1 and PES2 as seeds [59]. These sequences were used to search a compiled FASTA file containing the complete proteomes of selected eukaryotic species. Sequences above a specified inclusion threshold (E-value = 0.001) were retrieved and redundant sequences showing 100% identity were eliminated using CD-HIT [60]. The positive hits were aligned using MAFFT online with default settings, available at http://mafft.cbrc.jp/alignment/server/index.html (accessed on 9 September 2019) [61]. The resulting multiple sequence alignment (MSA) was used to generate a position-specific scoring table (hidden Markov model, hmm) using the hmmbuild tool from the HMMER suite. This profile was used again against the complied FASTA file, using hmmsearch. Positive hits were aligned again and used for the generation of a new hmm file, re-starting the whole cycle. This process was repeated until convergence, at which point no new information was obtained when a new data-mining cycle was done. The final MSA was trimmed using BMGE v1.2 (Block Mapping and Gathering with Entropy), with the following optional arguments: -m BLOSUM45 -h 0.6 -g 0.5:0.3 -b 3, and used for phylogeny reconstruction [62]. This was performed by the Maximum Likelihood method using PhyML v3.0, with the LG model, default settings and 500 bootstraps [63]. Final tree editing was performed using iTOL (accessed on 24 October 2019) [64].

The analysis of conserved domains was performed with NCBI Conserved Domain Database (https://www.ncbi.nlm.nih.gov/Structure/cdd/wrpsb.cgi (accessed on 10 June 2024)). Subcellular localization was analyzed using PredAlgo (https://lobosphaera.ibpc.fr/cgi-bin/predalgodb2.perl?page=main (accessed on 6 June 2024)), an algorithm specifically trained with green algal proteins. The presence of transmembrane regions was studied with the programmes DeepTMHMM (https://dtu.biolib.com/DeepTMHMM (accessed on 6 June 2024)), SOUSI (https://harrier.nagahama-i-bio.ac.jp/sosui/mobile/ (accessed on 6 June 2024)) and CCTOP (https://cctop.ttk.hu/). Hydrophobicity analysis was performed with Peptide 2.0 (https://www.peptide2.com/N_peptide_hydrophobicity_hydrophilicity.php (accessed on 4 June 2024)).

### 4.4. DNA Isolation and PCR Mapping

DNA isolation was performed as described by Sambrook et al. [65]. The control strain cc-5325 and the mutant strains *pesα* and *pesβ* were genotyped by PCR using 0.25 µM of oligonucleotide primers targeting the genomic sequence and primers flanking the left and right borders of the paromomycin-resistance cassette, CIB1 (Table S2). PCR reactions for *PESα* amplification contained 0.05 U·µL^−1^ GoTaq G2 Hot Start Polymerase from Promega (cat. no. M7401; Madison, WI, USA) and were performed under the following conditions: holding stage, 95 °C for 5 min; cycling stage, 40 cycles at 95 °C for 30 s (melting), 50 °C for 20 s (annealing) and 72 °C for 2 min (extension), with a final extension cycle at 72 °C for 5 min. PCR reactions for *PESβ* amplification contained 0.015 U·µL^−1^ KAPA HiFi DNA Polymerase from KAPA Biosystems, Inc. (cat. no. 7958897001; Wilmington, MA, USA) with 1× KAPA HiFi GC buffer, and were run using the following parameters: holding stage, 95 °C for 5 min; cycling stage, 40 cycles at 95 °C for 30 s (melting), 62 °C for 20 s (annealing) and 72 °C for 2 min (extension), with a final extension cycle at 72 °C for 5 min. PCR products obtained from *pesα* and *pesβ* mutants were cloned into pCR4 Blunt TOPO vector from Thermo Fisher Scientific (cat. no. 450245; Waltham, MA, USA), following the manufacturer’s instructions. DNA sequencing was performed by Macrogen Facility (https://dna.macrogen.com/ (accessed on 28 September 2021)).

### 4.5. RNA Isolation, cDNA Synthesis and Quantitative PCR Analyses

The harvested cells were lysed by incubating them with 70 mM Tris-HCl, pH 8, 200 mM NaCl, 20 mM EDTA, pH 8, and 2.7% SDS, at room temperature for 30 min [16]. Total RNAs were extracted with phenol:chloroform:isoamyl alcohol (25:24:1), as previously described [16]. Copy DNA (cDNA) was synthesized using MMLV reverse transcriptase (RT) from Thermo Fisher Scientific (cat. no. 28025013; Waltham, MA, USA) and an oligo-dT primer on 1 µg of total RNA as a template, following the manufacturer’s instructions. The cDNA was diluted to a final volume of 50 µL and 2 µL were used for quantitative PCR (qPCR). The Fast Universal SYBR Green Master mix from Millipore Sigma (cat. no. 04913914001; Burlington, MA, USA) was employed, using an Applied Biosystems StepOneTM Real-Time PCR System from Thermo Fisher Scientific (Waltham, MA, USA). The standard amplification program was used. The nucleotide sequences of the specific primers for qPCR analysis of *DGAT3*, *DGAT1*, *PDAT* and *GBLP* were previously reported [16]. The oligo sequences for *PESα* and *PESβ* are detailed in Table S2. Data was analyzed using LinRegPCR v11.0, a program for determining PCR efficiency and calculating starting concentrations of target sequences [66]. To ensure the comparability of Ct values despite differences in expression levels, we used template concentrations that showed acceptable linearity (R^2^ ≥ 0.98). Furthermore, only genes with amplification efficiencies between 1.8 and 2.2, and with less than 10% efficiency differences between each other were compared [67].

### 4.6. Lipid Extraction and Thin-Layer Chromatography

Lipid extraction was performed according to the method described by Bligh and Dyer [68]. Briefly, cells were resuspended in 200 µL of phosphate-buffered saline and vortexed for 20 s. Total lipids were extracted with 700 µL of chloroform:methanol (1:2, *v*/*v*) followed by overnight incubation at −20 °C. For phase partitioning, 233 µL of chloroform and 200 µL of H_2_O were added, samples were vortexed for 20 s, and the tubes were centrifuged at 1000× *g* for 20 min at room temperature. Successful lipid extraction was confirmed by the white color of the protein solid phase, indicating complete partitioning of chlorophyll into the lower chloroform phase, which was transferred to a new tube. Lipid extracts were dried under a nitrogen stream and resuspended in 30 µL of chloroform. For the resolution of TAGs and wax esters/FAPEs, lipid extracts were spotted onto 500-µm silica gel G-60 TLC plates (20 × 15 cm) under a nitrogen stream. A mixture of *n*-hexane:diethyl ether (96:4, *v*/*v*) was used as the mobile phase. Plates were pre-equilibrated for 10 min in a tank containing the solvent mixture and then developed until the solvent front reached 4 cm from the top. After drying the TLC plates for 10 min, lipid bands were visualized under blue light following spraying with 0.03% 2,7-dichlorofluorescein in methanol and exposure to ammonia vapor. Lipid classes were identified by comparison with co-spotted standards. Olive oil and jojoba oil were used as TAG and wax ester/FAPE standards, respectively. Oleic acid (product code MATSOL 101 OD; MATERIA Hnos SACIF, Mar del Plata, Argentina) was kindly provided as a free fatty acid standard. Commercial phytol from Sigma, which is a mix of cis-/trans-isomers (W502200; St Louis, MI, USA), was used as a standard. TAGs and wax esters/FAPEs were eluted from the silica with chloroform:methanol:water (5:5:1, *v*/*v*) and partitioned by adding 0.8 volumes of 1 N ammonia (for TAGs) or 0.8 volumes of water (for wax esters/FAPEs and phytol) to recover them in the organic phase. Each organic fraction was dried under a nitrogen stream. Purified TAGs were resuspended in 25 µL of isopropyl alcohol, and their total quantity was determined using an enzymatic assay (GPO-PAP method, TG Color, Wiener Lab, (Catalog number 1780111; Ciudad Autónoma de Buenos Aires, Argentina) following the manufacturer’s instructions. Wax ester/FAPE fractions were further analyzed by gas chromatography–mass spectrometry (GC–MS) to detect FAPE molecular species.

### 4.7. Detection of FAPEs by GC-MS

For identification and quantification purposes, the FAPE standard (phytylpalmitate) was prepared and purified as previously described [69]. GC-MS analyses were performed in full-scan mode (*m*/*z* 50–800) for identification, as well as in selected ion-monitoring (SIM) mode for quantification. The wax ester/FAPE fractions were analyzed on a GC-MS Agilent GC8890-MSD5977C system from Agilent Technologies (Santa Clara, CA, USA), which was equipped with a split/splitless injector operated in splitless mode and an HP-5MS GC (Hewlett-Packard/Agilent, Waldbronn, Germany).

### 4.8. Quantification of Phytol by HPLC

For phytol separation, lipid extracts were co-spotted with a commercial phytol standard onto silica gel H 60 TLC plates under a nitrogen stream. Plates were developed using a mobile phase of benzene:ethyl acetate (19:1, *v*/*v*) [70]. Lipid bands were visualized by spraying with 0.03% 2,7-dichlorofluorescein in methanol and exposing the plate to ammonia vapor. The phytol band was identified by co-migration with the standard, scraped from the silica, and eluted with chloroform:methanol:water (5:5:1, *v*/*v*). Dried phytol samples were reconstituted in methanol and analyzed using an Agilent 1260 Infinity HPLC system equipped with an Eclipse Plus C18 reverse-phase column (100 × 4.6 mm, 3.5 µm spherical particles, octadecylsilane-coated; Agilent Technologies; Waldbronn, Germany), as reported by Narai-Kanayama, with the following modifications [71]. Methanol was used as the mobile phase, delivered by a quaternary pump at 40 °C. The flow rate was 0.5 mL min^−1^. Phytol elution was monitored at 210 nm using a UV detector. An external calibration curve was constructed using standard solutions of phytol in the range of 0.5–16 μg mL^−1^ in methanol.

### 4.9. Chlorophyll Quantification

The harvested cells were resuspended in 1 mL of 80% (*v*/*v*) acetone and vortexed for 1 min. The extracts were then heated at 100 °C for 1 min and centrifuged at 6000× *g* for 5 min at room temperature. Successful chlorophyll extraction was confirmed by the white color of the pelleted cellular debris. The supernatant was transferred to a new tube for chlorophyll quantification. Absorbance measurements were taken at 663 nm (chlorophyll *a*) and 646 nm (chlorophyll *b*), and their concentrations were calculated using the equations described by Lichtenthaler [72]. The entire procedure was performed under dim light to avoid chlorophyll bleaching.

## 5. Conclusions

We demonstrate that *PESα* and *PESβ* are transiently induced by high light in parallel with TAGs. In addition, *PESα* knockdown results in overexpression of DGAT3, which may be functionally linked to PESα, along with TAG accumulation, under low light. We propose that PESα plays a role in light-dependent TAG production, but not in FAPE synthesis, in *C. reinhardtii* cells grown under light conditions in which phytol levels are not increased above basal levels. A follow-up to our work will involve analyzing the enzymatic activity of PESα and its regulatory mechanisms.

## Data Availability

Dataset available upon request from the authors.

## Supplementary Materials

The following supporting information can be downloaded at: https://www.mdpi.com/article/10.3390/plants14193044/s1, Figure S1: Multiple sequence alignment performed with MAFFT of PESα and PESβ from *C. reinhardtii* and PES1 and PES2 from *A. thaliana*; Figure S2. Knockdown of *PESα* and *PESβ* does not affect growth rate but increases cell area in the *pesα* mutant under standard light conditions. Table S1: Sources of the proteome files used for DAGAT superfamily biocomputational HHMER iterative profiling; Table S2: qPCR and PCR oligonucleotides [73,74,75,76,77,78,79,80,81,82,83,84,85,86,87,88,89,90,91,92,93,94,95,96].
