# Supplementary material for: Knock Down of Chlamydomonas reinhardtii Phytyl Ester Synthase α Triggers DGAT3 Overexpression and Triacylglycerol Accumulation Under Low-Light Conditions
Source: Plants (Basel). 2025 Oct 1;14(19):3044. doi: 10.3390/plants14193044 (PMC12526585; doi:10.3390/plants14193044)
Supplement: Supplementary file 1 [file plants-14-03044-s001.zip › Figure S1.pdf]

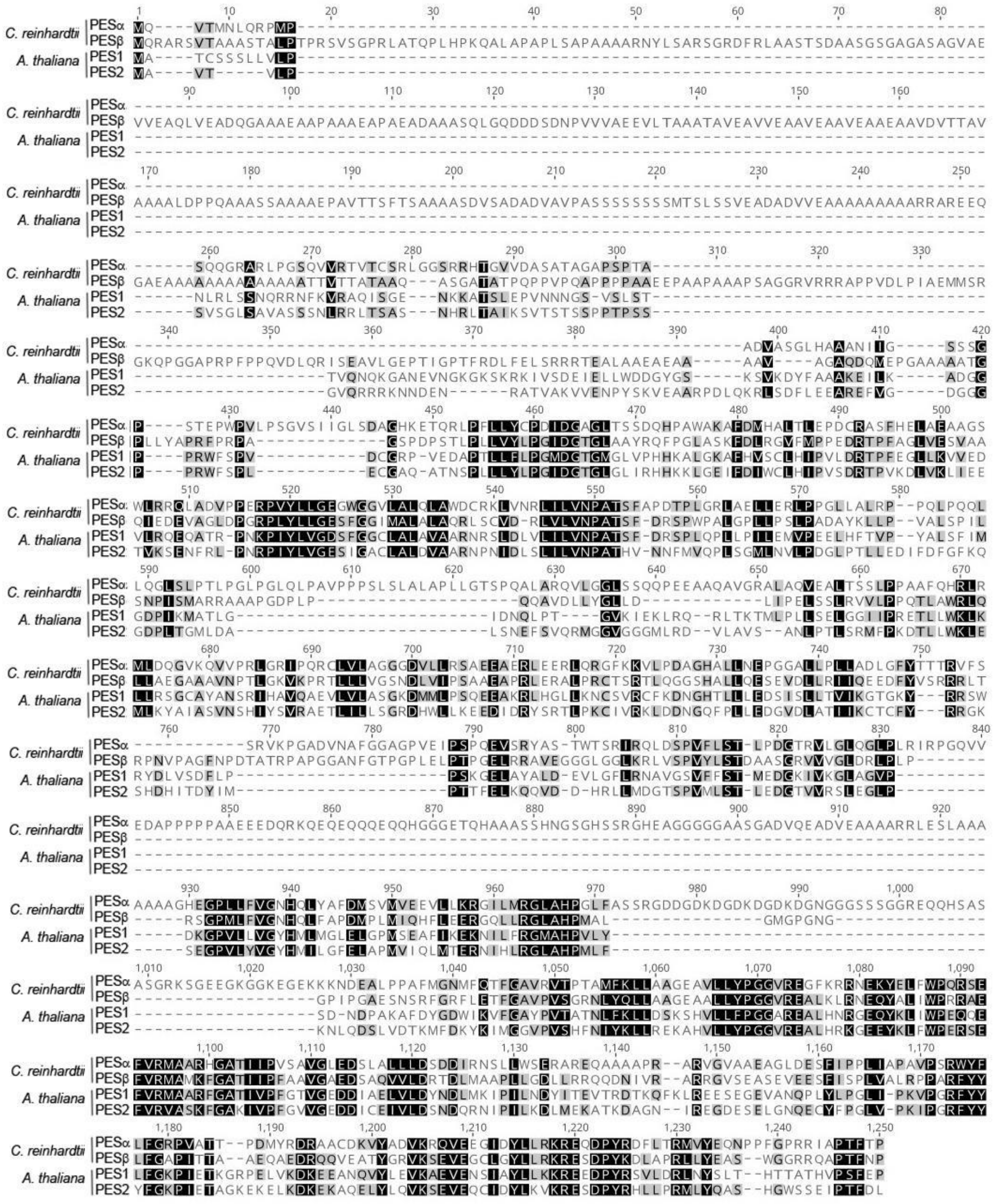

Figure S1. Multiple sequence alignment of PESα and PESβ from *C. reinhardtii* and PES1 and PES2 from A. thaliana. The alignment was performed with MAFFT using BLOSUM62 and default settings.
