# Supplementary material for: Knock Down of Chlamydomonas reinhardtii Phytyl Ester Synthase α Triggers DGAT3 Overexpression and Triacylglycerol Accumulation Under Low-Light Conditions
Source: Plants (Basel). 2025 Oct 1;14(19):3044. doi: 10.3390/plants14193044 (PMC12526585; doi:10.3390/plants14193044)
Supplement: Supplementary file 1 [file plants-14-03044-s001.zip › Figure S2.pdf]

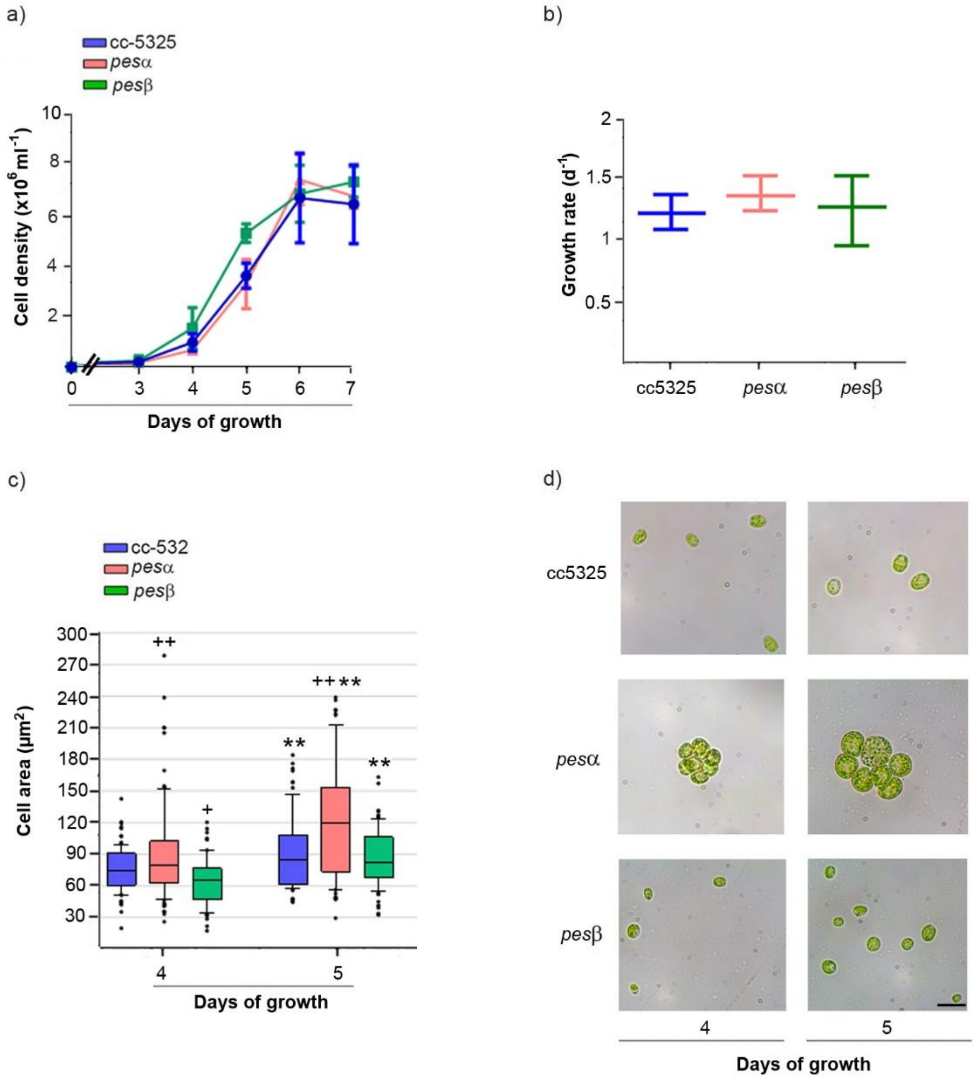

Figure S2. Knockdown of *PESα* and *PESβ* does not affect growth rate but increases cell area in the *pesα* mutant under standard light conditions. Cells from the parental cc-5325 (control), *pesα* and *pesβ* strains were grown in TAP medium under continuous light (50 µmol photons $m^{-2} s^{-1}$). Samples were harvested at 3, 4, 5, 6, and 7 days of growth. **(a)** Cell density was determined by cell counting with a hemocytometer. Vertical bars and symbols represent standard deviation and means, respectively (n=3). **(b)** Growth rates (d^{-1}) were calculated using the formula $\mu = (\ln(x_2) - \ln(x_1)) / (t_2 - t_1)$, where x_2 and x_1 are the cell densities at 6 and 3 days of growth, and t_2 (6 days) and t_1 (3 days) correspond to the end and beginning of the exponential growth phase, respectively [58]. Vertical bars indicate minimum and maximum values and horizontal strips indicate the median (n=3). **(c)** Cell area of cells harvested at 4 and 5 days of growth was determined using ImageJ 1.54g (Wayne Rasband, NIH, USA). Vertical bars indicate minimum and maximum values and horizontal black strips indicate median values (n=80). Significant differences between cc-5325 and *pesα*/*pesβ* mutants are indicated by plus symbols (+), while significant differences between 4 and 5 days of growth within each strain are indicated by asterisks (*) according to one-way ANOVA, post hoc Dunnett's: ++ ***P* < 0.05, + **P* < 0.1. **(d)** Representative images of cells harvested at 4 and 5 days of growth. Scale bar = 10 µm.
